# ASSOCIATIONS BETWEEN DISABILITY, CHRONIC DISEASES, AND DEPRESSIVE SYMPTOMS IN VIETNAMESE OLDER ADULTS

**DOI:** 10.1093/geroni/igad104.0845

**Published:** 2023-12-21

**Authors:** Christina Miyawaki, Joshua Garcia, Luis Medina, Kim Nguyen, Oanh Meyer, Kyriakos Markides

**Affiliations:** University of Houston, Houston, Texas, United States; University of Houston, Houston, Texas, United States; University of Houston, Houston, Texas, United States; University of Houston, Houston, Texas, United States; University of California at Davis, Davis, California, United States; University of Texas Medical Branch, Galveston, Texas, United States

## Abstract

After the fall of Saigon in 1975, several waves of Vietnamese immigrants/refugees migrated to the U.S. currently comprising 2.2 million individuals, the 4th-largest Asian-origin subgroup in the U.S. Despite the large number and their traumatic, adverse lifelong experiences, health research on this population is scarce. To fill this gap, we developed the Vietnamese Aging and Care Survey and collected health data on older Vietnamese in Houston, Texas, the 2nd-largest Vietnamese-populated metropolitan area in the nation. The purpose of this study was to examine the associations between their physical disability, chronic diseases, and depressive symptoms. Respondents (N=210) were 76 years old (mean), married (63%), and female (56%) with 8 years of education. They lived in the U.S. for 25 years (mean) and spoke Vietnamese only (88%) in multi-generation (80%) low-income households (91%, ≤$25K). They self-rated their health as fair/poor (80%) with ≥1 chronic condition (76%). Those with higher depressive symptoms were less likely to be married (p=0.004) and have more chronic diseases (p=0.004) compared to those without depressive symptoms. Regression analyses showed significant associations between arthritis (β=2.73), liver disease (β=6.24), ADL disability (β=0.72), and higher depressive symptoms. Limited control over their basic function (ADL disability) would restrict their daily lives, and mobility issues and pain (arthritis and liver disease) might impact them psychologically. While older Vietnamese may leverage their multi-generation living condition, and receive tangible support with ADL disability within their families, healthcare professionals should connect them with culturally and linguistically relevant adult daycare centers that provide social and exercise opportunities.

